# Aragonite-Polylysine: Neuro-Regenerative Scaffolds with Diverse Effects on Astrogliosis

**DOI:** 10.3390/polym12122850

**Published:** 2020-11-29

**Authors:** Tzachy Morad, Roni Mina Hendler, Eyal Canji, Orly Eva Weiss, Guy Sion, Refael Minnes, Ania Hava Grushchenko Polaq, Ido Merfeld, Zvy Dubinsky, Elimelech Nesher, Danny Baranes

**Affiliations:** 1 Department of Molecular Biology, Ariel University, Ariel 4070000, Israel; tzachy11@gmail.com (T.M.); roni.orany123@gmail.com (R.M.H.); eyal.canji@gmail.com (E.C.); orly1990@hotmail.com (O.E.W.); anethava@gmail.com (A.H.G.P.); mido@ivory-sw.com (I.M.); elimelechn@ariel.ac.il (E.N.); 2 School of Zoology, Faculty of Life Sciences, Tel Aviv University, Tel Aviv 69978, Israel; guy.sion@mail.huji.ac.il; 3 Institute for Land Water and Society, Charles Sturt University, P.O. Box 789, Elizabeth Mitchell Drive, Albury, NSW 2642, Australia; 4 Department of Physics, Ariel University, Ariel 4070000, Israel; refaelm@ariel.ac.il; 5 The Mina & Everard Goodman Faculty of Life Sciences, Bar-Ilan University, Ramat-Gan 5290002, Israel; dubinz@mail.biu.ac.il; 6 Institute for Personalized and Translational Medicine, Ariel University, Ariel 4070000, Israel

**Keywords:** aragonite, polylysine, coral skeleton, astrocytic reactivity, neural migration

## Abstract

Biomaterials, especially when coated with adhesive polymers, are a key tool for restorative medicine, being biocompatible and supportive for cell adherence, growth, and function. Aragonite skeletons of corals are biomaterials that support survival and growth of a range of cell types, including neurons and glia. However, it is not known if this scaffold affects neural cell migration or elongation of neuronal and astrocytic processes, prerequisites for initiating repair of damage in the nervous system. To address this, hippocampal cells were aggregated into neurospheres and cultivated on aragonite skeleton of the coral *Trachyphyllia geoffroyi* (Coral Skeleton (CS)), on naturally occurring aragonite (Geological Aragonite (GA)), and on glass, all pre-coated with the oligomer poly-D-lysine (PDL). The two aragonite matrices promoted equally strong cell migration (4.8 and 4.3-fold above glass-PDL, respectively) and axonal sprouting (1.96 and 1.95-fold above glass-PDL, respectively). However, CS-PDL had a stronger effect than GA-PDL on the promotion of astrocytic processes elongation (1.7 vs. 1.2-fold above glass-PDL, respectively) and expression of the glial fibrillary acidic protein (3.8 vs. and 1.8-fold above glass-PDL, respectively). These differences are likely to emerge from a reaction of astrocytes to the degree of roughness of the surface of the scaffold, which is higher on CS than on GA. Hence, CS-PDL and GA-PDL are scaffolds of strong capacity to derive neural cell movements and growth required for regeneration, while controlling the extent of astrocytic involvement. As such, implants of PDL-aragonites have significant potential as tools for damage repair and the reduction of scar formation in the brain following trauma or disease.

## 1. Introduction

Biomaterials are a key tool in regenerative medicine and tissue engineering. Many of them are biocompatible, cell adhesive, and supportive, and successfully applied for growth of a wide range of tissues including osseous, cardiac, skeletal muscles, and neuronal [1,2,3,4,5]. Aragonite, a polymorph mineral of calcium carbonate, is formed naturally in high pressure metamorphic rocks and can be found in geological sediments [6]. Aragonite has also a biological origin; it is the main molecule in marine skeletons and shells. The skeletal aragonite is produced and deposited by the marine animals by the action of specific enzymes that generate micro crystalline structures in specific calcification centers, followed by their accumulation into fibrous aragonitic constructs [7]. The coralline aragonite is cell adhesive, supports cell growth in vitro [8,9,10] and has been used as bone replacement in animals [11,12,13] and humans [14,15].

Coating coralline biomaterials with adhesive polymers guarantees successful cell binding as well as elevated levels of cell survival and growth. The oligomer poly-D-lysine (PDL) is a cationic α-carbon synthetic polymer of the amino acid lysine that, at pH 7, contains highly dense positively charged hydrophilic amino groups and, due to its D chiral type of lysine, is resistant to enzymatic degradation and has prolonged cell adherence [16]. Being positively charged, PDL can bind to different types of surfaces and proteins [17] and has been utilized to coat different types of culture products to improve cell attachment [18]. PDL enhances adherence of various cell types [19] including neurons and glia [20,21]. Indeed, PDL-coated coralline scaffolds (CS-PDL) are superior matrices for the cultivation of neural cells from the hippocampi of postnatal rat brains [8,22,23,24,25,26]. Moreover, CS-PDL scaffolds are permissive and supportive for cultivated hippocampal tissue [23]. Hence, the CS-PDL composite is a scaffold with strong and supportive interactions with nervous tissue.

The effects of aragonite scaffolds that have been shown on astrocytes are of great relevance to damage repair in the central nervous system (CNS). Astrocytes are involved in tissue repair by undergoing astrogliosis—a spectrum of astrocytic behavior that includes morphologic modifications and increase motility [27,28]. Astrogliosis occurs in traumatic brain injury (TBI), ischemic damage, and neurodegenerative diseases and has both beneficial and harmful influence on nervous tissue recovery. While it can lead to blood–brain barrier repair and secretion of neuro-protecting molecules which support neuronal development and maturation, it can interfere with recovery by exacerbating inflammation, causing synapse phagocytosis, axon growth interference, and neuronal development inhibition [29]. An additional central event in astrogliosis is up-regulation of the cytoskeletal glial fibrillary acidic protein (GFAP), a hallmark of astrocyte reactivity, and astrocytic hypertrophy [30]. GFAP is up regulated upon CNS trauma or other neuro-pathologies [31] and, when dimerized with Vimentin [32], can reduce tissue regeneration. For example, GFAP/Vimentin double knockout mice have better synaptic regeneration in the hippocampus [33], better axonal regeneration of the optic nerve after crush injury [34], and improved regeneration and functional recovery after spinal cord injury trauma [35], as compared to wild type. One additional burden to recovery produced by astrogliosis is that it generates scar tissue. Reactive astrocytes can aggregate into a scar at the wound borders to form a barrier and reduced axonal growth [36,37,38]. Hence, controlling astrocytic activity and motility are essential goals that should be taken into consideration when designing treatments for CNS trauma [39,40,41,42].

One of the ways to identify treatments that regulate astrogliosis is by assessing the effect of biomaterials on astrocytic dynamics and reactivity in appropriate in vitro systems, such as neurosphere culture. Neurospheres are spheroid structures formed by association of a few hundred neural cells under specific culture conditions [43,44]. As a 3D structure with tight cell contacts, neurospheres mimic the natural, in vivo environment of neurons and glia better than the planar, 2D, dissociated cultures, in terms of differentiation, morphology, growth, proliferation, and migration [22,23]. Neurosphere cultures have been used to test efficacy of 3D scaffolds made of hydrogels [45], polyethylene terephthalate track-etched membranes [46], 3D synthetic polyethylene glycol, and gelatin-based scaffolds [47]. The neurosphere culture is suitable for measurements of neuronal cell motility and outgrowth since when neurospheres encounter a solid substance, they break down and the neural cells migrate out away from the neurosphere core [48]. Such directional growth is convenient for quantification of cell migration and process outgrowth.

In this study, we analyzed the influence of PDL-aragonite scaffolds on the migration and process outgrowth of hippocampal neurons and astrocytes grown in neurospheres in vitro. Two aragonite-PDL composites were tested: A skeleton of the coral *Trachyphyllia geoffroyi* (CS-PDL) and naturally-occurring geological aragonite (GA-PDL). We demonstrate that CS-PDL and GA-PDL enhance cell migration and axonal sprouting similarly, but CS-PDL is a stronger activator of astrocytic process elongation and GFAP expression, compared to a control of glass-PDL, probably due to its rougher surface. Hence, PDL-aragonite scaffolds can be engineered to simultaneously initiate nervous tissue recovery while controlling the level of astrogliosis, introducing a new opportunity for treating damage repair in the CNS following trauma or disease.

## 2. Materials and Methods

### 2.1. PDL-Aragonite Matrix Preparation

*Trachyphyllia geoffroyi* exoskeleton and geological aragonite (GA, Alfa Aesar, Lancashire, United Kingdom) crystals were treated for the removal of organic residues. The samples were soaked in hypochlorite acid (35%, 10 min; Romical, Beer Sheva, Israel) and then exposed to NaOH solution (1 M, 5 min, Romical, Beer Sheva, Israel). Next, they were exposed to hydrogen peroxide (40%, 10 min; Romical, Beer Sheva, Israel). The sanitized samples were washed, dried and then ground using Smart Dentin Grinder (KometaBio, Fort Lee, NJ, USA). The grains were then sifted using a 40 μm sieve. A solution was made using coral grains under 40 μm and double distilled water (DDW, 25 mg coral grains/mL). The solution was deposited onto coverslips (12 mm, Menzel-Glaser), heated to 80 °C until dried, and then autoclaved. The coverslips were then covered with 20 µg/mL PDL (30–70 kD, A-003-M, Sigma-Aldrich, St. Louis, MO, USA) in DDW, incubated overnight at 4 °C, washed with DDW and air dried.

### 2.2. Fourier-Transform Infrared Spectroscopy (FTIR)

FTIR measurements were carried out using an FTIR spectrometer (Jasco, 6800 FV, Tokyo, Japan) equipped with a Diamond ATR device (Jasco, ATR Pro One, Tokyo, Japan). The effective dimensions of the diamond are 1.8 mm diameter. The refractive index of the diamond is 2.4 and the angle of incidence in our device was 45 degree, generating 1 reflection. The calculated depth of penetration is ~2 μm. The radiation from the IR source of the spectrometer was focused into the ATR diamond and the output radiation (from the other side of the diamond) was focused onto a DLaTGS (Deuterated Lanthanum α Alanine doped TriGlycine Sulphate) detector. Powder samples were placed on the diamond ATR and pressure (700 kg/cm^2^) was applied to produce better contact between the sample and the diamond. To trace the PDL in the samples, we compared the samples’ spectra with the spectrum of PDL powder. Measurements were carried out in the spectral range of 650–4000 cm^−1^. Each spectrum was an average of 64 scans to increase the signal to noise ratio (SNR). The spectra were analyzed using Spectra Analysis^TM^ (Jasco, Tokyo, Japan).

### 2.3. Neurosphere Cell Culture

Neural primary cells were extracted from postnatal rat hippocampi according to Baranes, et al. [26]. Concisely, hippocampi were extracted and dissociated using trypsin (0.25% 30 min, 37 °C; Sigma-Aldrich, St. Louis, MO, USA). Then, the cells were triturated in a recovery medium consisting of Minimal Essential Eagle’s medium (87%, Sigma-Aldrich, St. Louis, MO, USA), heat-inactivated fetal bovine serum (10%, Sigma-Aldrich, St. Louis, MO, USA), D-Glucose (2%, Thermo Fisher Scientific, Waltham, MA, USA) and L-Glutamine (1%, Thermo Fisher Scientific, Waltham, MA, USA).

Association of the cells into neurospheres was performed according to Marshall, G.P, et al. [49]. The primary neural cells (10^5^ cells/mL^−1^) were divided over a 24 wells plate, with 1 mL proliferation culture medium per well. The medium consisted of Minimal Essential Eagle’s medium (45%, Sigma-Aldrich, St. Louis, MO, USA), Dulbecco’s Modified Eagle medium (40%, Sigma-Aldrich, St. Louis, MO, USA), Nutrient mixture F-12 Ham (10% (w/v); Sigma-Aldrich, St. Louis, MO, USA), D-Glucose (0.75%, Thermo Fisher Scientific, Waltham, MA, USA), B-27 (0.5%, Thermo Fisher Scientific, Waltham, MA, USA), bovine serum albumin (0.25%, Sigma-Aldrich, St. Louis, MO, USA), L-Glutamine (0.25%, Thermo Fisher Scientific, Waltham, MA, USA), and epidermal growth factor (20 ng/mL; Thermo Fisher Scientific, Waltham, MA, USA). Cells were incubated (37 °C, 10% CO_2_) for one week, to allow assembly into neurospheres.

The neurospheres were then counted and distributed onto coverslips, 20–40 neurospheres per coverslip, (previously coated with poly-D-lysine (30–70 kD, 20 µg/mL; A-003-M, Sigma-Aldrich, St. Louis, MO, USA) overnight and washed with DDW) with 100 µL the supplemented culture medium, Minimal Essential Eagle’s medium (45%, Sigma-Aldrich, St. Louis, MO, USA), Dulbecco’s Modified Eagle medium (40%, Sigma-Aldrich, St. Louis, MO, USA), Nutrient mixture F-12 Ham (10%, Sigma-Aldrich, St. Louis, MO, USA), D-Glucose (0.75%, Thermo Fisher Scientific, Waltham, MA, USA), B-27 (0.5%, Thermo Fisher Scientific, Waltham, MA, USA), bovine serum albumin (0.25%, Sigma-Aldrich, St. Louis, MO, USA), L-Glutamine (0.25%, Thermo Fisher Scientific, Waltham, MA, USA), kynurenic acid (0.01%, Sigma-Aldrich, St. Louis, MO, USA) and 0.01% anti-mitotic treatment composed of 70% uridine (Sigma-Aldrich, St. Louis, MO, USA) and 30% fluoro-deoxy-uridine (Sigma-Aldrich, St. Louis, MO, USA), overnight. Then, 0.5 mL of supplemented culture medium was added, and the cells were incubated (37 °C, 10% CO_2_) for 7 days, until fixation.

### 2.4. Transmission Electron Microscopy

Preparation of biological samples and Araldite blocks is described in Jeger, et al. [50]. The blocks were sectioned (60–90 nm thick) with a diamond knife, using a Leica Ultracut UCT microtome (Leica Microsystems, Nussloch, Germany), and picked up on 300-mesh copper grids. The sections were contrasted by uranyl acetate (Sigma-Aldrich, St. Louis, MO, USA) and lead citrate (Sigma-Aldrich, St. Louis, MO, USA) and observed under the JEOL JEM-1230 TEM (JEOL Ltd., Tokyo, Japan) operated at 80 kV. Electron micrographs were taken using TemCam-F214 (Tietz Video & Image Processing Systems (TVIPS), Gauting, Germany).

### 2.5. Scanning Electron Microscopy

Cells were fixed for 30 min (37 °C) in 2% paraformaldehyde (Sigma-Aldrich, St. Louis, MO, USA) and 2.5% glutaraldehyde (Sigma-Aldrich, St. Louis, MO, USA) in 0.1 M phosphate buffer (Biological Industries, Kibbutz Beit-Haemek, Israel), rinsed twice with PBS for 10 min, and then placed in increasing concentrations of ethanol (50, 75, 90, 95% v/v ethanol Romical, Israel, in distilled H_2_O) 5 times for 15 min each, and three times for 10 min each in absolute ethanol (Romical, Beer Sheva, Israel). Samples were rinsed in a series of different hexamethyldisilazane (HMDS; Sigma-Aldrich, St. Louis, MO, USA) concentrations (33.3, 50, 66.6% v/v in 100% ethanol) and three times in 100% HMDS for 1 min each, glued to mounting slabs, coated with gold, and observed through a Quanta 200ESEM/SEM (FEI).

### 2.6. Immunofluorescence

Samples were fixed using paraformaldehyde solution (PFA, 4%, Sigma-Aldrich, St. Louis, MO, USA). The fixed samples were permeabilized with Triton×100 (0.25%, TEDIA, Fairfield, OH, USA). Next, the cells were immersed in blocking solution containing Triton×100 (1%) and inactivated normal goat serum (3%). The samples were incubated with the following antibodies, overnight: Anti-Neurofilament Medium (NFM, Cat#Ab9034, Abcam, Cambridge, England) polyclonal, rabbit, 0.002 mg/mL, for marking axons. Anti-Glial Fibrillary Acidic Protein (GFAP, Cat#Ab7260, Abcam, Cambridge, England) polyclonal, rabbit, 0.002 mg/mL, for marking astrocytes. Lastly, the cells were washed and stained with secondary antibodies goat-anti- mouse/rabbit Alexa 488/555 (Millipore, Burlington, MA, USA) and 4′,6-diamidino-2-phenylindole (DAPI, Sigma- Aldrich, St. Louis, MO, USA, marks DNA for the visualization of cell nuclei). The coverslips were then mounted on slides, mounted with ProLong™ Diamond Antifade Mountant (Thermo-Fisher Scientific, Waltham, MA, USA) and stored in 4 °C.

### 2.7. Image Analysis

Neurospheres were examined using an Axiovert microscope (Zeiss, Oberkochen, Germany) equipped with Plan Fluor lens EC PlnN × 5/0.16, EC PlnN × 10/0.3 and LD PlnN × 40/0.6 with Fs49 (365/445, DAPI) and Fs20 (546/575, Alexa 555) filters. Image analysis was performed using Fiji software [51].

Fluorescent intensity was measured by drawing an outline marking a field with cells, and calculating the area, mean grey value and integrated density. The background intensity was measured in the same manner. The corrected total cellular fluorescence (CTCF) was measured using the equation: Integrated density-(area of selected cell × mean fluorescence of background readings). Cell count was done using the “Find maxima” function after manually removing the neurospheres’ core. Migration distance as well as length of astrocytic processes were measured using the segmented line manually marking the distance from the core/from the end of the cell body. Axons, as well as the number of processes spreading out of neurosphere were measured using Sholl analysis function.

### 2.8. Statistics

#### 2.8.1. Data Analysis

Student’s *t*-test was used, if the variables did not significantly depart from a normal distribution according to a one sample Kolmogorov–Smirnov test. Otherwise, a Mann–Whitney U-test was used. We also used Pearson correlation. Statistical analyses of the present study were performed using SPSS software (version 22.0; IBM Corp., Armonk, NY, USA). The probability, set at α = 0.05, was two-tailed. Although one tailed was enough for test improvement. Results presented with mean 95% confidence intervals (*SD*); * *P* < 0.05; ** *P* < 0.01; *** *P* < 0.001. ns = non-significant.

#### 2.8.2. Normal Distributions

All data were tested for normality (Kolmogorov–Smirnov test). For the distribution of the variable, the average number of migrating cells per experiment did not significantly depart from a normal distribution according to one sample Kolmogorov–Smirnov test: *N* = 17, Mean = 532.76 ± SD = 389.13, *Z* = 0.914, *D* = 0.222, *P* = 0.486. For the variable migration distance (μm), *N* = 14, Mean = 447.86 ± SD = 185.77, *Z* = 0.837, *D* = 0.224, *P* = 0.486. For the mean extension length of astrocytes (GFAP) in different slides, *N* = 91, Mean = 130.83 ± SD = 47.88, *Z* = 1.573, *D* = 0.165, *P* = 0.014. Thus, we used a Mann–Whitney U-test rather a student’s *t*-test. For the mean gray value, *N* = 9, Mean = 12.83 ± SD = 8.28, *Z* = 0.848, *D* = 0.283, *P* = 0.469. For maximal axon length, *N* = 14, Mean = 0.928 ± SD = 0.916, *Z* = 1.021, *D* = 0.273, *P* = 0.248.

## 3. Results

**Figure 1:**
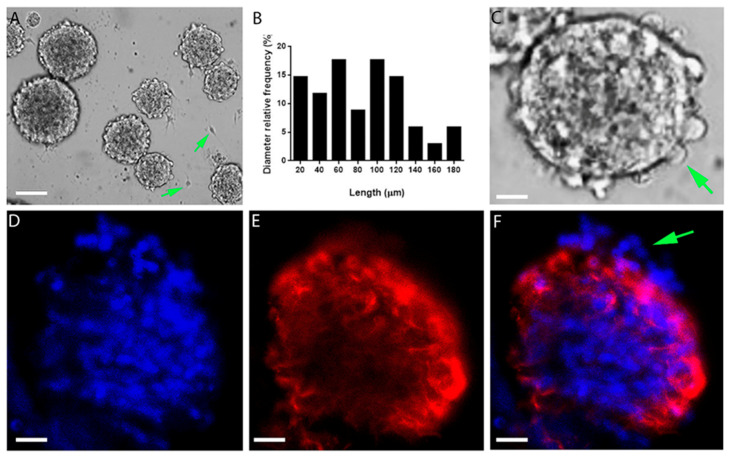
Astrocytes are confined to the periphery of suspended neurospheres. (**A**) Neurospheres in suspension. Arrows point to cells growing out of the neurospheres. (**B**) Distribution of neurosphere diameters. (**C**) Individual cells protruding from the main structure (example indicated by the green arrow). (**D**–**F**) Cell nuclei (DAPI, blue), GFAP (red). Arrow in (**F**) indicates a cell protruding from the surface. Scale: (**A**) — 100 μm; (**C**) — 20 μm; (**D**–**F**) — 25 μm.

To understand if aragonite-PDL matrices affect neural cells’ migration and process outgrowth, the neurosphere type of hippocampal cell culture was utilized. In this culture system, the cells are packed and confined in spheres and any cell growth or migration out of the sphere can be easily detected and quantified. Neurospheres were generated by cultivating dissociated hippocampal cells in suspension, in culture dishes with low adhesive properties. The cells aggregated under these conditions, forming ball-like clusters of dozens to hundreds of cells (Figure 1A), with diameters of 20–180 μm (Figure 1B). Occasionally, individual cells emerged from the neurosphere edge (arrow in Figure 1C, see also arrow in Figure 1D), but cell growth out of the neurospheres was rare (arrows in Figure 1A). Within the neurospheres, cells were in the inner and peripheral regions (Figure 1D), as shown by the nuclear marker DAPI. By contrast, astrocytes and their processes were concentrated predominantly in the periphery, as revealed by immunostaining of GFAP (Figure 1E,F).

**Figure 2:**
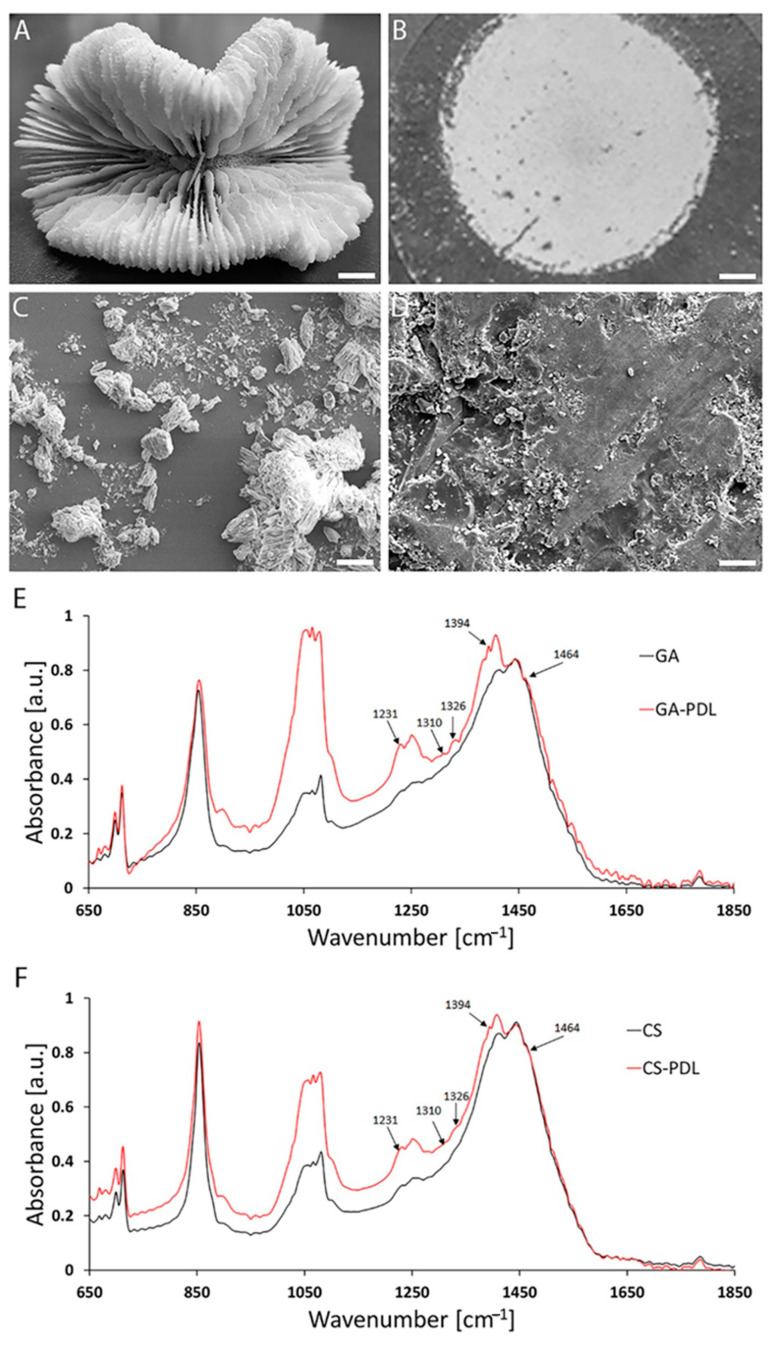
*Trachyphyllia geoffroyi* (CS) and aragonite (GA) have different surface roughness and they both bind poly-D-lysine (PDL). (**A**) The skeleton of the coral *T. geoffroyi.* (**B**) A glass coverslip covered with the coral skeleton grains. (**C**) SEM of the coral skeleton grains. (**D**) SEM of GA. (**E**,**F**) FTIR spectra of GA and CS, respectively. Characteristic PDL peaks are indicated by arrows. Scale: (**A**) — 2 mm; (**B**) — 120 μm. (**C**,**D**) — 20 μm.

**Figure 3:**
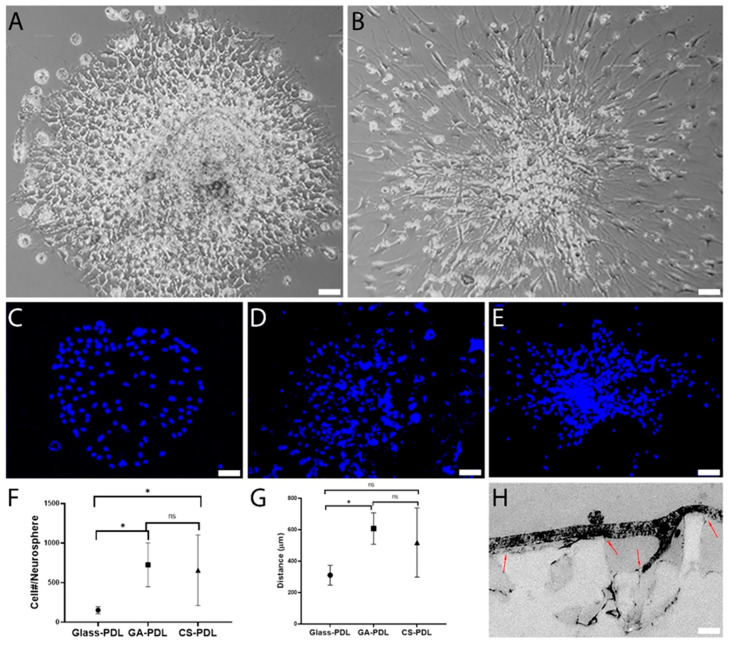
Enhanced neural cell migration on geological aragonite (GA-PDL) and PDL-coated coralline scaffolds (CS-PDL). 7-day-old cultures: (**A**) Glass-PDL. (**B**) CS-PDL. (**C**–**E**) DAPI of glass-PDL (**C**), GA-PDL (**D**), CS-PDL (**E**). (**F**) Number of migrating cells per neurosphere. Student’s *t*-test: Glass-PDL N = 5, Mean = 152.00 ± 44.67, GA-PDL *N* = 6, Mean = 725.50 ± 276.87, *t*_5.311_= −4.996, *P* = 0.003, CS-PDL N = 6, Mean = 657.33 ± 445.11, *t*_5.121_ = −2.764, *P* = 0.039. GA-PDL vs. CS-PDL: *t*_10_ = 0.319, *P* = 0.757. (**G**) Maximal migration distance. Student’s *t*-test: Glass-PDL *N* = 6, Mean = 310.00 ± 63.24, GA-PDL *N* = 3, Mean = 606.66 ± 100.16, *t*_7_ = −5.546, *P* = 0.001, CS-PDL *N* = 5, Mean = 518.00 ± 220.38, *t*_9_ = −2.226, *P* = 0.053. GA-PDL vs. CS-PDL: *t*_6_ = 0.642, *P* = 0.544. (**H**) TEM of a neural cell interacting with the tips of CS-PDL grains (arrows). Scale: (**A**,**B**) — 50 μm; (**C**,**D**) — 30 μm. (* *P* < 0.05; ns = non-significant).

To monitor migration of cells out of the neurospheres in response to PDL-aragonite scaffolds, the neurospheres must be shifted from their suspended configuration to an adherent one. To achieve this, the skeleton of the coral *Trachyphyllia geoffroyi* (Figure 2A) was ground and attached to glass coverslips (Figure 2B). In parallel, a similar procedure was performed with GA grains (not shown). Both aragonite matrices were then coated with PDL. Scanning electron micrographs of the two scaffolds on the coverslips showed a broad range of sizes of CS grains (from a few nanometers to 40 μm) and had a rough and complex surface (Figure 2C). In contrast, the GA grains were larger with smoother surfaces (Figure 2D). PDL binding to the GA and CS was verified by detecting its characteristic peaks under Fourier-transform infrared spectroscopy (FTIR) spectrum analysis (Figure 2E,F). The neurospheres were then seeded, where they attached themselves to the surface of the aragonites and collapsed within minutes. This was followed by cell spreading radially from the neurosphere core (Figure 3A,B). Labeling cell nuclei with DAPI revealed that while on glass, PDL cell spreading was approximately uniform around the center (Figure 3C), on GA-PDL and CS-PDL it was irregularly shaped and appeared to extend further (Figure 3D,E). The average number of migrating cells per neurosphere was 725.50 ± 276.87 and 657.33 ± 445.11 for GA-PDL and CS-PDL, respectively, with no statistical differences (Figure 3F). However, the average number of migrating cells on glass-PDL was more than fourfold lower (152/neurosphere). Similarly, the average maximal migration distance was about two-fold larger on CS-PDL and GA-PDL (460 µm) than on glass-PDL (267 µm) (Figure 3G). Transmission electron microscopy showed that neural cells interacted with the coralline aragonite surface by stretching between the tips of the grains (Figure 3H). Thus, interaction of neural cells with the tips of the grains of the aragonite matrices accelerates their migration compared to their migration rate on glass.

**Figure 4:**
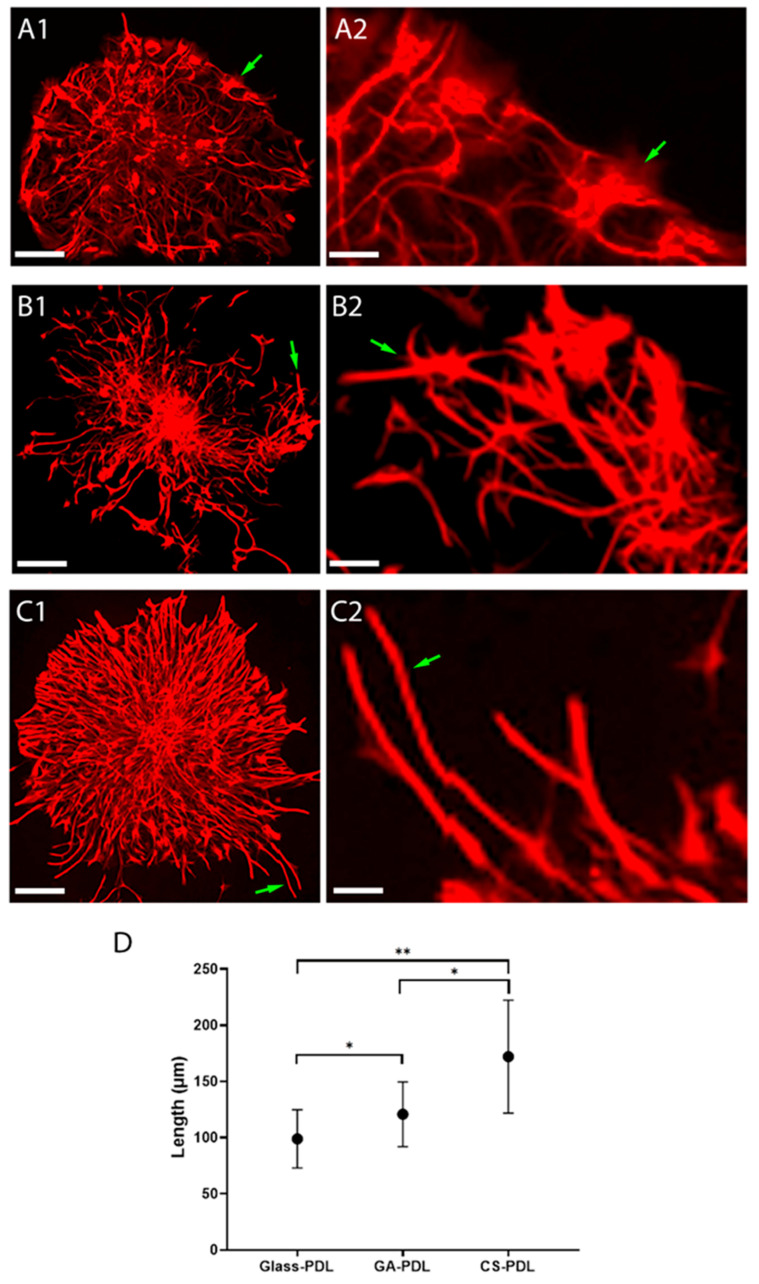
CS-PDL promotes elongation of astrocytic processes more robustly as compared to GA-PDL. GFAP immunofluorescence. (**A1**,**A2**) glass-PDL. Green arrows indicate flat astrocytes. (**B1**,**B2**) GA-PDL. (**C1**,**C2**) CA-PDL. Green arrows in both indicate spiky astrocytes. (**D**) Maximal length of processes. Pearson correlation: *N =* 91, *r =* 0.634, *P <* 0.001. **Scale**: (**A1**–**C1**) 60 mm; (**A2**–**C2**) 15 mm. (**D**) Mean extension length of astrocytes (glial fibrillary acidic protein (GFAP)) in different slides. Mann–Whitney U-test: Glass-PDL *N* = 31, Mean = 98.93 ± 25.94, GA-PDL *N* = 29, Mean = 120.85 ± 28.80, *Z* = −2.729, *P* = 0.006, CS-PDL *N* = 31, Mean = 172.06 ± 50.24, *Z* = −3.942, *P* < 0.001. Pearson correlation: *N* = 91, *r* = 0.634, *P* < 0.001. GA-PDL vs. CS-PDL: *Z* = −3.942, *P* < 0.001. * *P* < 0.05; ** *P* < 0.01.

**Figure 5:**
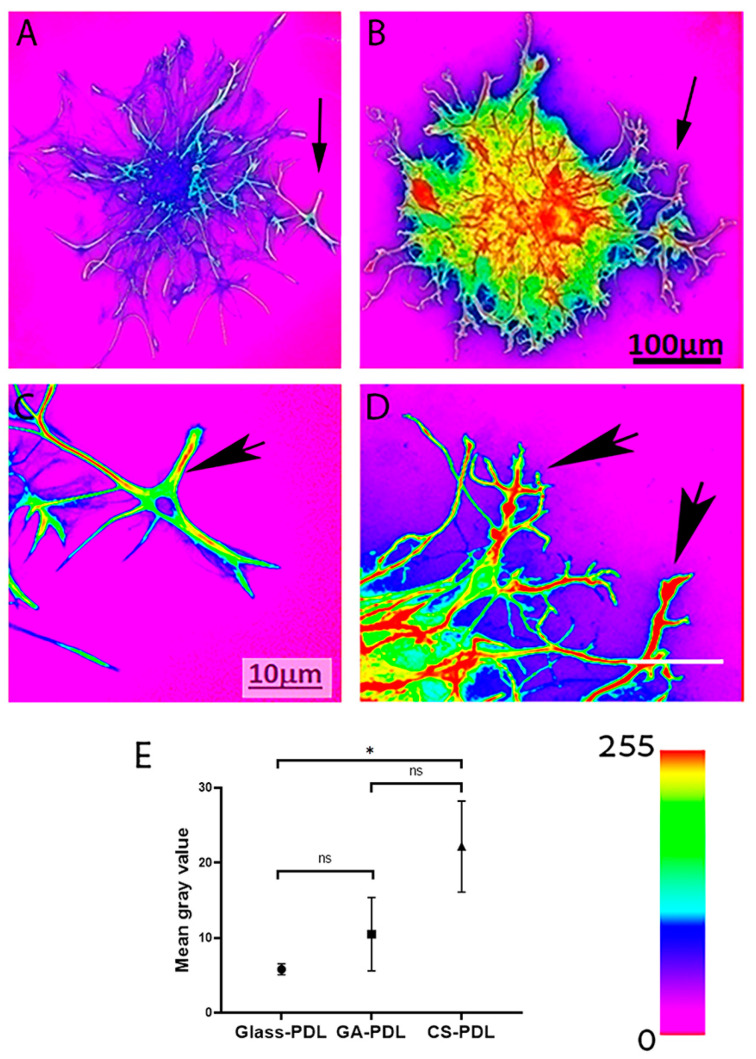
CS-PDL elevates GFAP expression in migrating astrocytes. Color-coded GFAP fluorescent images. (**A**) Glass-PDL. (**B**) CS-PDL. Arrows point to enlargement areas (**C**,**D**), respectively. Arrows in (**C**,**D**) point to growth cones. (**E**) Quantification of GFAP expression level. Student’s *t*-test: Glass-PDL *N* = 3, Mean = 5.81 ± 0.74, GA-PDL *N* = 3, Mean = 10.49 ± 4.89, *t*_2.092_ = −1.640, *P* = 0.237, CS-PDL *N* = 3, Mean = 22.18 ± 6.06, *t*_2.060_ = −4.639, *P*_Two-tailed_ = 0.041. GA-PDL vs. CS-PDL: *t*_4_ = −2.597, *P*_Two-tailed_ = 0.060. Scale: (**A**,**B**) — 100 μm; (**C**,**D**) — 10 μm. (* *P* < 0.05; ns = non-significant).

The next set of experiments, shown in Figure 4 and Figure 5, was designed to understand if aragonite matrices influence two parameters of astrogliosis, process lengthening and GFAP expression. Astrocytic processes on glass-PDL cultures were of two configurations—thin and flat (Figure 4(A1,A2)). By contrast, astrocytic on the CS-PDL and the GA-PDL cultures were spiky with predominantly elongated, thin processes (Figure 4(B1,B2,C1,C2)), typical of activated astrocytes. The length of the astrocytic processes was also sensitive to the presence of the two aragonite matrices, but in a differential way. The length reached a maximal average value of 99 μm on glass-PDL and was significantly bigger on GA-PDL (121 μm) but was even more elongated on CS-PDL (172 μm). Thus, by contrast to their similarity in promoting cell migration, CS-PDL has a robust effect, promoting the elongation of astrocytic processes 40% more than GA-PDL.

Astrocytes also reacted to contact with the aragonite matrices by elevating GFAP expression levels. The first evidence for this is the presence of primarily GFAP expressing astrocytes on GA-PDL and CS-PDL cultures (Figure 4(B1,C1) and Figure 5B), in contrast to glass-PDL cultures which contain two astrocytic cell populations, expressing high or low GFAP levels (Figure 4(A1,A2) and Figure 5A). Color-coding of the GFAP images reveled that the level of this protein was higher in the processes (Figure 5A,B) and growth cones (Figure 5C,D) of astrocytes in the presence of CS-PDL. Image analysis revealed that CS-PDL, but not GA-PDL, increased the average GFAP level by 1.73-fold, compared to GA-PDL and glass-PDL. Thus, CS-PDL is an enhancer of GFAP expression in the migrating astrocytes in contrast to GA-PDL.

**Figure 6:**
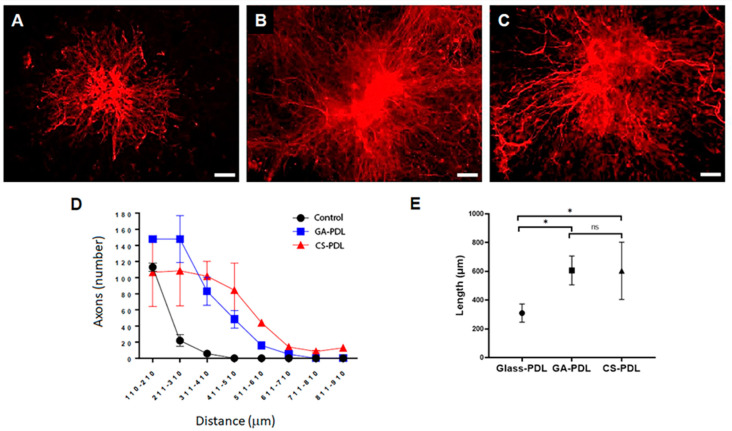
GA-PDL and CS-PDL promote axonal sprouting. Neurofilament M (NFM) immunofluorescence. (**A**) Glass coverslip. (**B**) GA-PDL. (**C**) CS-PDL. (**D**) Sholl analysis of axonal length. (**E**) Axonal maximal length. Glass-PDL *N* = 6, Mean = 310.00 ± 63.24, GA-PDL *N* = 3, Mean = 606.67 ± 100.16, *t*_7_ = −5.546, *P* = 0.001, CS-PDL *N* = 5, Mean = 604.00 ± 198.82, *t*_9_ = −3.451, *P* = 0.007. GA-PDL vs. CS-PDL: *t*_6_ = 0.021, *P* = 0.984. Scale: (**A**–**C**) — 100 µm. (* *P* < 0.05; ns = non-significant).

The above results raised the question whether the effect of the aragonite matrices is specific to astrocytes. To address this, axons sprouting from neurons in cultivated neurospheres were visualized by immunofluorescence of the protein neurofilament M (NFM). Extensive axonal sprouting (100–150/neurosphere) was detected in both aragonite matrices and the control glass cultures (Figure 6A–C). However, in GA-PDL and CS-PDL cultures, the extent of sprouting was higher (Figure 6D). At 210–500 μm from the neurosphere core, the number of axons extending out of the neurospheres grown on CS-PDL and GA-PDL was 5–20-fold higher than on glass-PDL. Moreover, whereas the average maximal axonal length on GA-PDL and on CS-PDL were similar (607 μm vs. 604 μm, respectively), it was significantly higher (1.95-fold) than that measured on glass-PDL (Figure 6E). Thus, GA-PDL and CS-PDL are equally potent inducers of axonal outgrowth.

## 4. Discussion

This work demonstrates that when the naturally occurring GA and the biologically derived CS aragonite minerals are positively charged by PDL they are equally robust promoters of neural cell motility and outgrowth, but CS-PDL has a greater effect in the activation of astrocytes. This difference, which as discussed below, may be attributed to dissimilarities in the physical properties of the scaffolds, and can be of clinical relevance. The characterization of these properties provides a new means to design aragonite implants with pre-determined control over astrocytic activity, which, as mentioned in the introduction, has positive and negative roles in wound healing in the injured brain.

This work expands and generalizes the previously published roles of aragonite minerals as neuro-promotive substances in three ways: First, in addition to the known ability of coralline aragonite to protect and promote growth of hippocampal neural cells and tissue in culture [52], we show here that it also influences neural cell migration and process outgrowth. Second, the skeleton used here is of a different coral than those used in earlier publications, suggesting that the neuro-supportive effects of the coralline aragonites are general and not coral species-specific. Third, the fact that GA, a non-biological aragonite, is effective, suggests that the neuro-regenerative capacity of aragonite is independent of the mineral origin. Thus, aragonite minerals, probably of any source, are superior as substances for maintenance, growth and motility of neural cells in vitro as compared to the classical neural culture substrate—glass.

However, although aragonite by itself is a strong cell adhesive [8,53], boosting its cell supportive capacity required the presence of PDL. This requirement raises the question as to whether GA-PDL and CS-PDL are stronger than glass-PDL simply because they bind PDL more efficiently than glass. Apparently, the FTIR results shown in Figure 2E,F support this possibility, as PDL was detected on GA and CS but not on glass (not shown), probably because the level on glass was below the detection level of the equipment. But if GA and CS have similar PDL association, what is the explanation for their differential influence on astrocytic action? It seems that additional parameters, related to the aragonite physical and chemical properties, should be considered. Indeed, cultivation of neuronal cells in the absence of PDL can be achieved, although of reduced quality, on aragonite matrices but not on glass (not shown). Therefore, stronger PDL binding is unlikely to be the mechanism through which GA and CS gain superiority over glass. Their calcium carbonated content and surface properties, in conjunction with PDL, should be considered, as further discussed below.

PDL was chosen because it is the classical substrate for hippocampal cell culture. However, it may be toxic for treatment in vivo due to its D chiral amino acid oligomers which cannot be enzymatically cleaved. A more biocompatible polymer, poly-L-lysine, which has also been used as substrate for neuronal culture [54,55] may have reduced toxicity, but probably would be less stable. Polymers with other types of amino acids, such as poly-arginine [56] and poly-ornithine [48,57], may prove beneficial in vivo. Other bioactive, non-amino acid, positive polymers, such as chitosan and alginate [58] may accelerate the functions of aragonite. Thus, it would be logical and of clinical importance to fabricate aragonite scaffolds with diverse polymer coating.

As mentioned above, despite being both coated by PDL, CS-PDL has a more robust effect than GA-PDL on the elongation of astrocytic process and is the only of the two that promotes elevation in GFAP expression (Figure 4E and Figure 5E). What could be the basis for such differences? One possibility is that CS binds more PDL molecules or more efficiently than the GA. The likelihood of this is small, considering the chemical identity and IR spectra similarity between GA-PDL and CS-PDL. An alternative explanation is based on the difference in the surface roughness between the two aragonites. As shown in Figure 2C,D, the CS matrix is rougher that the GA. It has been reported that astrogliosis in culture can be affected by surface topography. For example, astrocytes aligned along nano grooves of different sizes showed inverse correlation between the grooves size and the level of GFAP RNA [59]. Similarly, rat cortical astrocytes grown on a nano-porous anodic aluminum oxide substrate with a pore size of 20 nm expressed significantly lower GFAP levels than on pore sizes of 90 nm [60]. Recently, we demonstrated that coralline scaffolds of distinct surface topologies differentially affect the distribution and GFAP levels of reactive hippocampal dissociated astrocytes [23] and tissue [22] in vitro. While astrocytes grown on CS grains elevate their GFAP and form cell clusters, astrocytes on intact pieces of coral skeleton, with 140 μm diameter pores, are non-homogenously distributed and have reduced capacity to cluster. Thus, the higher level of surface roughness of CS-PDL may explain its ability to elevate GFAP in astrocytes, an effect which GA cannot affect. This discrepancy is of great importance for damage repair. It provides an opportunity to design neuro-regenerative implants with some level of control over astrogliosis, as further detailed in the discussion summary.

In contrast to the different effects on astrocytes, GA-PDL and CS-PDL have a similar effect on neural cell migration and axonal sprouting, suggesting that these processes are surface-roughness independent. Hence, the chemical rather than structural nature of CS and GA is probably the accelerator of migration and axonal growth. It is possible that the capacity of neural cells to absorb calcium ions from a coralline matrix, as has been shown [24], is the mechanism that underlies the effect of CS-PDL and GA-PDL on neural cell motility and axonal growth.

In summary, this work is the first to demonstrate regulation of motility and outgrowth of neural cells by aragonite scaffolds, findings of significant implications for regenerative medicine of the CNS. The similarities and discrepancies between GA-PDL and CS-PDL yields a set of new considerations to be taken into account when designing an appropriate aragonite scaffold implant for CNS wound repair: The stiffness of the aragonite scaffold required to mobilize and sprout the neural cells and their processes. In addition, the scaffold surface adherence and shape are of great therapeutic relevance. The surface must be coated with an adhesive polymer and its roughness pre-designed to control the level of astrocytic activity and thereby of astrogliosis and scar formation. The surface can be designed by selecting aragonite grain sizes and/or by mixing CS and GA grains in defined ratios. Hence, we may conclude that fabricating aragonite-PDL matrices possessing all or partial combinations of the above properties would yield a novel family of scaffolds that can tune neuronal activation and astrogliosis to different levels. Equipped with such innovative capacity, implants made of pre-engineered aragonite-PDL would be superior to existing ones, having higher rate of success in repairing brain damage and restoring lost tissue following injury, stroke, and neurodegenerative diseases.
